# Oncoplastic Surgery Versus Total Breast Reconstruction in Locally Advanced and Multicentric Carcinomas. A Matched Comparative Cohort Study of Oncologic and Aesthetic Outcomes

**DOI:** 10.1002/jso.70372

**Published:** 2026-08-17

**Authors:** Aline Regina Nunes‐Reis, Regis Resende Paulinelli, Rosemar Macedo Sousa Rahal, Luiz Fernando Jubé Ribeiro, Ruffo de Freitas Júnior

**Affiliations:** ^1^ Federal University of Goias Goiania Brazil; ^2^ Araujo Jorge Cancer Hospital Goiania Brazil; ^3^ Albert Einstein Israeli Hospital Goiania Brazil

**Keywords:** extreme oncoplasty, mastectomy, multicentric breast cancer, oncologic outcomes

## Abstract

**Background/Objective:**

This study aimed to compare extreme oncoplastic surgery (EO‐partial breast reconstruction for tumors > 5 cm or multicentric lesions) with extreme reconstruction (ER‐mastectomy with reconstruction for tumors >5 cm or multicentric lesions).

**Methods:**

This retrospective cohort study evaluated clinical and pathological parameters, complications, recurrence, survival, and aesthetic outcomes in 276 women, with large or multicentric breast carcinoma who underwent breast reconstruction between March 2004 and September 2025 at a private oncoplastic practice and two tertiary hospitals in Goiânia, Brazil. Data were analyzed using SPSS (version 15.0), with statistical significance set at *p* < 0.05.

**Results:**

Mean follow‐up was 44 months for EO and 57 months for ER (*p* = 0.03). Clinical tumor size before chemotherapy was 60 mm in both groups; after neoadjuvant chemotherapy, tumors were smaller in EO (37 mm vs. 44.5 mm, *p* = 0.02). EO required fewer procedures, with 74% completed in a single surgery, while ER more often required multiple operations (*p* < 0.01). No significant differences were found between groups regarding margin status or re‐excision rates. In the ER group, 51.40% of patients developed complications, with 18.60% requiring reoperation due to severe events (*p* < 0.01). Five‐year overall survival was similar (94.40% EO vs. 97.50% ER, *p* = 0.53), as was locoregional recurrence‐free survival (97%). Patient‐reported outcomes and physician assessments demonstrated superior satisfaction and aesthetic results in EO.

**Conclusions:**

EO provides equivalent oncologic safety to ER, with fewer surgeries, lower complication rates, and improved aesthetic and psychosocial outcomes. These findings support EO as a valid alternative to mastectomy with reconstruction in selected patients with large or multicentric breast cancer.

**Abbreviations**
BCS: breast‐conserving surgery
BMI: Body mass index
CT: chemotherapy
DCIS: ductal carcinoma in situ
EO: extreme oncoplasty
ER: extreme reconstruction
IDC: invasive ductal carcinoma
IHC: Immunohistochemistry
ILC: invasive lobular carcinoma
LD: Latissimus dorsi
LICAP: Lateral intercostal artery perforator
MRI: Magnetic resonance imaging
OBCS: Oncoplastic breast‐conserving surgery
PROM: patient‐reported outcome measure
TRAM: Transverse rectus abdominis myocutaneous

## 1 Introduction

Oncoplastic breast‐conserving surgery (OBCS) integrates the principles of oncology and reconstructive surgery to achieve both safe oncological outcomes and aesthetically pleasing results [1]. Oncoplasty addresses some limitations of traditional breast‐conserving surgery (BCS) by enabling larger resection, less positive margins, minimizing deformity, and achieving superior aesthetic outcomes [1, 2]. As a result, oncoplastic surgery enables BCS for many women who would otherwise require mastectomy to be treated with segmental resection [3].

Oncoplastic BCS enables the maintenance of high patient satisfaction rates, even in cases of large or multicentric tumors traditionally considered candidates for mastectomy due to potential challenges in achieving acceptable cosmetic outcomes. Notably, women undergoing OBCS report significantly higher satisfaction with overall breast appearance, the nipple‐areolar complex, treatment outcomes, and psychological well‐being compared to those undergoing mastectomy [4, 5, 6].

The efficacy of conservative treatment for early‐stage lesions is well‐established by randomized studies provided clear surgical margins are achieved, but its effectiveness for large or multicentric tumors is still controversial [7, 8, 9, 10, 11, 12]. The majority of evidence supporting extreme oncoplasty derives from small retrospective studies [5, 6, 10, 11, 13, 14]. These studies typically compare extreme oncoplasty to standard breast‐conserving surgery [5, 11]. However, in clinical practice, the primary alternative to extreme oncoplasty for patients with large or multicentric tumors is often mastectomy, with or without reconstruction. This study distinguishes itself by including one of the largest cohorts with long‐term follow‐up, thereby providing a more robust evidence base to guide clinical decision‐making.

We hypothesized that EO surgery provides equivalent oncologic safety and superior aesthetic and quality‐of‐life outcomes compared to total breast reconstruction, after mastectomy. This retrospective matched cohort study encompasses a comprehensive dataset, including patient and tumor characteristics, a large variety of surgical techniques, complication rates, number of procedures, local recurrence rates and overall survival with long‐term follow‐up, patient‐reported quality of life, and both subjective and objective assessments of breast symmetry.

## 2 Patients and Methods

### 2.1 Patient Selection

This study included 276 women diagnosed with large or multicentric breast carcinoma. Surgeries were performed between March 2004 and September 2025 at the private practice of an oncoplastic breast surgeon and at two tertiary hospitals (Clinicas Hospital and Araújo Jorge Cancer Hospital) in Goiânia, Brazil. Patients were matched 1:1 by age and date of surgery, with a maximum variation of 5 years.

To ensure consistency and reproducibility, this study adopted a strict and objective criteria for lesion classification. Multicentric tumors were defined as those exhibiting a distance greater than 2 cm between tumor foci. Extreme lesions were characterized as an in situ or invasive tumor exceeding 5 cm in size or exhibiting multicentricity.

All patients underwent either partial or total breast reconstruction for breast carcinomas. Surgical interventions were categorized as follows:

- *Extreme Oncoplasty (EO)*: Partial breast reconstruction, after breast conserving surgery, for in situ or invasive breast carcinomas larger than 5 cm or those with multicentricity, determined based on clinical or pathological evaluation, before or after neoadjuvant chemotherapy (CT);
- *Extreme Reconstruction (ER)*: Total breast reconstruction, after mastectomy, for in situ or invasive breast carcinomas larger than 5 cm or those with multicentricity, determined based on clinical or pathological evaluation, before or after neoadjuvant CT.

Inclusion criteria required completion of the reconstructive process and radiation therapy at least 6 months prior to the follow‐up consultation and did not plan to undergo further reconstructive procedures. No sample size calculation was performed, but the number of cases exceeds most of previously published cohorts.

### 2.2 Data Collection

This study employed a retrospective cohort design. Data on patient satisfaction, quality of life assessments, recurrence, and survival were collected prospectively from medical records and patient‐administered questionnaires. A dedicated form was used to obtain information regarding socioeconomic and cultural factors, tumor characteristics, surgical procedures performed, and patient satisfaction with the aesthetic outcome. Standardized frontal photographs were taken 6 months to 2 years post‐procedure (including post‐radiotherapy, when applicable). A 50 mm lens was used, with the images framed to include the shoulders and elbows. The study was conducted in accordance with the Declaration of Helsinki (2013 revision) [15] and was approved by the hospitals' Research Ethics Committee (No. 2.198.758). Informed consent was obtained from all participants.

### 2.3 Preoperative Assessment and Surgical Procedure

The choice between EO and ER was based on tumor characteristics, breast‐tumor ratio, patient preference, and surgeon expertise, reflecting real‐world decision‐making. Standard preoperative screening included mammography and breasts ultrasound. Magnetic Resonance Imaging (MRI) was not routinely used in all cases. Indications followed criteria from the literature [16], however, the exam was frequently unavailable within the public healthcare services. In patients undergoing neoadjuvant CT, skin tattooing, metal clips or radioactive seeds were utilized as localization markers.

The best surgical option for each patient was determined after evaluating the breast‐tumor relationship, tumor location, degree of breast ptosis and glandular density. In general, volume displacement techniques—such as reduction mammoplasty, glandular or dermoglandular flaps—were employed in patients with large or ptotic breasts. In cases involving small, non‐ptotic breasts or tumors too large for mammoplasty techniques, volume replacement strategies were used either exclusively or in combination. These included fat grafting, perforator flaps, or myocutaneous flaps [17, 18, 19, 20, 21]. Mastectomy was reserved for situations in which an aesthetically acceptable result could not be achieved through oncoplastic techniques, according to the patient's expectations; in cases of high genetic or familial risk, or when preferred by the patient. Contralateral symmetry procedures were performed when necessary to achieve aesthetic balance and patient satisfaction.

Axillary access was achieved through the primary breast incision whenever feasible. When necessary, an axillary incision was employed. Sentinel node biopsy or axillary dissection was performed in accordance with the clinical guidelines applicable in the year of treatment [22].

Intraoperative frozen section analysis was not routinely performed for all surgical margin assessment or sentinel lymph node evaluation. In cases of positive surgical margins (residual tumor), re‐excision of the margins or mastectomy was performed to achieve clear margins in all cases.

### 2.4 Adjuvant Treatment and Follow‐Up

Patients received neoadjuvant and/or adjuvant systemic therapies, including endocrine therapy, CT, and radiotherapy, according to their TNM stage and current clinical guidelines. Postoperatively, patients were followed by the same surgical team. A structured follow‐up schedule was implemented: 6‐monthly visits for the first 5 years, followed by annual visits thereafter. Comprehensive surveillance included annual clinical examinations and appropriate imaging studies to monitor for local recurrence, regional recurrence, and distant metastases [22].

### 2.5 Methods for Evaluating Aesthetic Results

Patient satisfaction and quality of life were assessed using the BREAST‐Q Breast Reconstruction Postoperative Module [23, 24, 25]. The BREAST‐Q is a validated patient‐reported outcome measure (PROM) specifically designed to assess patient perceptions of outcomes following breast surgery. It comprehensively evaluates various aspects of health‐related quality of life impacted by breast cancer and breast reconstruction from the patient's perspective. Additionally, it has implications for research and improvement of clinical care, as it enables comparison of scores across different patient populations [23, 24]. Higher scores on the Breast‐Q indicate greater satisfaction and improved quality of life. The questionnaire includes domains evaluating psychosocial, physical, and sexual well‐being; satisfaction with breast appearance; and overall care experience [24]. The postoperative BREAST‐Q module consists of the following scales, namely Satisfaction with Breasts, Psychosocial Well‐being of the Chest, Sexual Well‐being, and Physical Well‐being, Satisfaction with Outcomes and Satisfaction with Care. The responses on each scale are summed and then transformed using Q‐Score (New York, NY; https://webcore.mskcc.org/breastq/scoring.html) to a scale of 0 (worst) to 100 (best).

The photographs were analyzed using BCCT.core software, which objectively evaluates the symmetry between breasts through measurements and differences in coloration [26]. The BCCT.core software was developed by the Breast Research Group at the University of Porto and categorizes the cosmetic outcome into one of four categories (excellent, good, fair, and poor) based on the scoring of color, shape, geometry, irregularity, and roughness of the visual appearance of the treated breast, compared to the untreated breast, using patient photographs [26]. Originally, it was created to evaluate the cosmetic outcome of breast‐conserving surgery [26]. Later, it was validated for the evaluation of breast reconstruction after mastectomy [27].

The main surgeons involved in the study (RRP) evaluated breast symmetry and aesthetic outcomes using the Harvard scale. This scale rates aesthetic results as follows: poor (severely distorted breast), fair (noticeably different but not severely distorted), good (slightly different), and excellent (nearly identical to the contralateral breast) [28]. Compared to the BCCT.core software, the Harvard scale demonstrated moderate concordance, with low inter‐observer variability and a stronger correlation with patient satisfaction [29].

The BREAST‐Q questionnaire and symmetry assessment were conducted between 6 months and 2 years following the final reconstructive surgery.

“Postoperative complications were assessed and graded according to the Clavien‐Dindo classification system, which stratifies surgical morbidity into five grades based on the severity of the complication and the type of intervention required [30].

### 2.6 Statistical Analysis

“Data were analyzed using SPSS statistical software (version 15.0). The Kolmogorov–Smirnov test was employed to assess the distribution of variables. Normally distributed numeric variables were compared using parametric tests. Non‐normally distributed numeric variables were compared using non‐parametric tests. Ordinal variables were compared using Mann–Whitney *U* test. Categorical variables were compared using the chi‐square test or Fisher's exact test, as appropriate. Statistical significance was set at *p* < 0.05.”

Patient medical records and national death registries were systematically reviewed in 2025 (with database lock on September 30) to update follow‐up information. Data collection encompassed local recurrence, contralateral breast cancer, distant metastasis, and mortality. Survival analyzes were performed to assess local recurrence and overall survival. Kaplan–Meier survival curves were generated, and differences between groups were evaluated using the log‐rank test. Multivariable analyzes were conducted employing Cox proportional hazards regression models to estimate hazard ratios and 95% confidence intervals.

## 3 Results

A total of 276 women diagnosed with advanced breast cancer were included in the study: 210 had T3 or T4 tumors (76.09%), 62 (22.46%) had multicentric tumors, and four (1.45%) patients presented with both locally advanced and multicentric disease. In terms of institutional origin, 93 (33.70%) were sourced from the Clinics Hospital of the Federal University of Goiás, 86 (31.20%) from one of the main oncoplastic surgeon's private offices, and 97 (35.10%) from the Araújo Jorge Cancer Hospital.

Table 1 shows the patients' tumors and treatments characteristics according to the surgical categories. The median follow‐up in the EO group was 44 months and in the ER group 57 months (*p* = 0.03). The median time since the last procedure was 33 months in the EO group and 31 months in the ER group (*p* = 0.60). There was no significant difference between the groups regarding mean age. The prevalence of smoking and diabetes was also similar between the two groups. Patients in the EO group exhibited a higher Body Mass Index (BMI) (*p* < 0.01). Among women in the ER group, there was a higher proportion of Caucasian individuals (69%, *p* = 0.01) and those covered by private health insurance (*p* < 0.01). The clinical tumor size prior to CT was 60 mm in both groups. However, the pathological tumor size was smaller in the EO group (*p* = 0.02). The median specimen weight in the EO group was 112 g, significantly lower than in the ER group. Invasive ductal carcinoma was the most frequent histological type across both groups, although patients in the ER group showed a higher prevalence of invasive lobular carcinoma and ductal carcinoma in situ (DCIS) (*p* < 0.01). According to the St. Gallen molecular classification, the most frequent subtype was Luminal A, with no significant differences observed between the groups (*p* = 0.42).

**Table 1:** Characteristics of patients, tumors, and clinical treatments.

	EO	ER	*p*
Age (mean ± SD)	54.25 (±10.72)	53.14 (±10.92)	0.40
BMI (mean ± SD)	27.88 (±3.99)	25.84 (±3.75)	**< 0.01**
Follow‐up (months)	44.00 (18.75–76.50)	57.00 (29.00–98.50)	**0.03**
Time since last procedure (months)	33.00 (13.00–65.00)	31.00 (11.00–63.00)	0.60
Caucasian	76 (55.07%)	96 (69.57%)	**0.01**
Private health insurance	27 (19.57%)	59 (42.75%)	**< 0.01**
Current smoker	7 (5.22%)	2 (1.45%)	0.081
Diabetes	14 (10.14%)	7 (5.07%)	0.11
Clinical tumor size (before CT)	60.00 (40.00–65.00)	60.00 (40.00–70.00)	0.15
Total pathological tumor size (invasive + in situ)	37.00 (14.50–60.00)	44.50 (20.75–70.00)	**0.02**
Specimen weight (g)	112.00 (55.00–336.25)	364.50 (258.25–589.25)	**< 0.01**
Clinical staging			0.26
0	0 (0.00%)	6 (4.5%)	
I	9 (6.52%)	7 (5.07%)	
II	42 (30.42%)	46 (33.33%)	
III	87 (63.04%)	79 (57.25%)	
Chemotherapy			
Neoadjuvant	77 (72.46%)	63 (45.99%)	0.17
Adjuvant	38 (27.54%)	40 (29.20%)	
Radiotherapy	138 (100%)	84 (60.87%)	**< 0.01**
Histology			**< 0.01**
IDC	119 (86.23%)	105 (77.21%)	
ILC	7 (5.07%)	11 (8.09%)	
DCIS	3 (2,17%)	16 (11.76%)	
Grade 3	29 (21.48%)	26 (19.40%)	0.67
St. Gallen classification			0.42
Luminal A	52 (38.52%)	45 (33.83%)	
Luminal B	30 (22.22%)	30 (22.56%)	
Luminal B/HER	25 (18.52%)	33 (24.81%)	
HER2+	8 (5.93%)	12 (9.02%)	
Triple negative	20 (14.81%)	13 (9.77%)	
Multicentric tumor	30 (21.74%)	36 (26.47%)	0.36

Abbreviations: BMI, Body mass index; CT, chemotherapy; DCIS, ductal carcinoma in situ; IDC, invasive ductal carcinoma; IHC, immunohistochemistry; ILC, invasive lobular carcinoma.

Table 2 shows that contralateral symmetrization was performed in over 70% of women in both groups (*p* = 0.68), while immediate reconstruction was more frequent in the EO group (89.86%; *p* = 0.02). The rates of positive and close surgical margins were similar between groups, as was the need for re‐excision. Conversion to mastectomy was required in three women (2.17%) in the EO group. Level II reconstruction was the most commonly employed technique for partial breast reconstruction (62.32%), whereas direct‐to‐implant reconstruction was the predominant approach for total breast reconstruction (42.75%). Most patients who underwent EO had a single surgical procedure (74.64%), while those who underwent ER more frequently required multiple surgeries (*p* < 0.01). Nipple–areola complex reconstruction was more common in the ER group (30.40%; *p* = 0.01). Intraoperative margin frozen section analysis was utilized in 59.80% of women in the EO group and in 37.90% of those in the ER group (*p* < 0.01). In the ER group, 51.40% of patients experienced complications, with 18.60% presenting severe complications that required reoperation (*p* < 0.01), classified as Clavien–Dindo grade II or III [30]. Complications were less frequent in the EO group.

**Table 2:** Surgical characteristics and outcomes.

*N* (%)	EO	ER	*p*
Immediate reconstruction	124 (89.86%)	107 (77.54%)	**0.02**
Contralateral symmetrization	102 (73.91%)	99 (71.74%)	0.68
Contralateral risk reducing mastectomy	1 (0.72%)	6 (4.35%)	0.06
Nipple and areola reconstruction	24 (17.39%)	42 (30.43%)	**0.01**
Number of procedures			**< 0.01**
1	103 (74.64%)	54 (39.13%)	
2	22 (15.94%)	46 (33.33%)	
3	7 (5.07%)	25 (18.12%)	
≥ 4	6 (4.34%)	13 (9.42%)	
Type of reconstruction			**< 0.01**
Level I	26 (18.84%)	0 (0.00%)	
Level II	86 (62.32%)	0 (0.00%)	
Level III (perforators)	21 (15.22%)	0 (0.00%)	
Expander	0 (0.00%)	32 (23.19%)	
Direct‐to‐implant	0 (0.00%)	59 (42.75%)	
LD with implant/expander	0 (0.00%)	3 (2.17%)	
Autologous LD	5 (3.62%)	7 (5.07%)	
TRAM flap	0 (0.00%)	37 (26.81%)	
Associated fat grafting	12 (8.70%)	26 (18.84%)	**0.01**
Frozen section	82 (59.85%)	52 (37.96%)	**< 0.01**
Margins			
Positive	11 (7.97%)	9 (6.52%)	0.64
< 1 mm	1 (0.76%)	4 (2.99%)	0.37
Reoperation for margins			
Re‐excision	9(6.52%)	5 (3.65%)	0.42
Mastectomy	3 (2.17%)	—	
Any complication	47 (34.06%)	71 (51.45%)	**< 0.01**
Severe complication (requiring reoperation)	8 (6.40%)	25 (18.66%)	**< 0.01**

Abbreviations: LD, Latissimus dorsi; TRAM, Transverse rectus abdominis myocutaneous.

As shown in Table 3, according to the BREAST‐Q assessment, patients in the EO group reported significantly higher satisfaction with their breasts and psychosocial well‐being compared to those in the total reconstruction group.

**Table 3:** Patient satisfaction and quality of life according to the postoperative BREAST‐Q.

Median (IQR)	EO	ER	*p*
Satisfaction with the breasts	76.50 (65.00–91.00)	69.00 (57.75–81.00)	**< 0.01**
Satisfaction with results	100.00 (86.00–100.00)	100.00 (75.00–100.00)	0.22
Psychosocial well‐being	86.00 (71.00–100.00)	82.00 (63.00–100.00)	**0.04**
Sexual well‐being	69.50 (52.50–99.75)	63.00 (49.00–90.00)	0.07
Physical well being	66.00 (57.00–77.00)	66.00 (57.00–77.00)	0.77
Satisfaction with the nipple	74.00 (62.50–96.50)	84.00 (50.00–97.25)	0.69
Satisfaction with information	80.00 (65.00–100.00)	77.50 (65.00–100.00)	0.51
Satisfaction with the reconstructive surgeon	100.00 (100.00–100.00)	100.00 (91.00–100.00)	0.36
Satisfaction with medical staff (others)	100.00 (100.00–100.00)	100.00 (100.00–100.00)	0.37
Satisfaction with non‐medical staff	100.00 (100.00–100.00)	100.00 (100.00–100.00)	

Abbreviations: EO, extreme oncoplasty; ER, extreme reconstruction.

According to Table 4, the majority of patients in both groups achieved good or excellent outcomes. However, based on the Harvard scale, the ER group had a higher incidence of poor results compared to the EO group (*p* < 0.01).

**Table 4:** The overall cosmetic scores for treated patients according to the Harvard scale and BCCT.core software.

	Harvard scale (medical opinion)				BCCT. core software		
Score	EO *N* (%)	ER *N* (%)	*p*		EO *N* (%)	ER *N* (%)	*p*
Poor	1 (0.72%)	6 (4.35%)	**< 0.01**		4 (2.90%)	2 (1.46%)	0.15
Regular	25 (18.12%)	29 (21.01%)			34 (24.64%)	29 (21.17%)	
Good	66 (47.83%)	80 (57.97%)			89 (64.49%)	88 (64.23%)	
Excellent	46 (33.33%)	23 (16.67%)			11 (7.97%)	18 (13.14%)	

Abbreviations: EO, extreme oncoplasty; ER, extreme reconstruction.

Table 5 indicates that, overall survival in 5 years was 94.40% in the EO group and 97.50% in the ER group (*p* = 0.53), while locoregional recurrence‐free survival in 5 years was 97% in both groups (*p* = 1.18). Overall survival and locoregional recurrence‐free survival rates were comparable between groups, as demonstrated by Kaplan–Meier curves (Figures 1 and 2).

**Table 5:** Overall survival and locoregional recurrence free survival in 5 years according to type of surgery.

	EO	ER	HR	CI 95%	*p*
Overall survival	94.47%	97.59%	1.61	(0.36–7.20)	0.53
Locoregional recurrence free survival	97.02%	97.72%	2.55	(0.64–10.20)	0.19

Abbreviations: CI, confidence interval; EO, extreme oncoplasty; ER, extreme reconstruction; HR, hazard Ratio.

**Figure 1:**
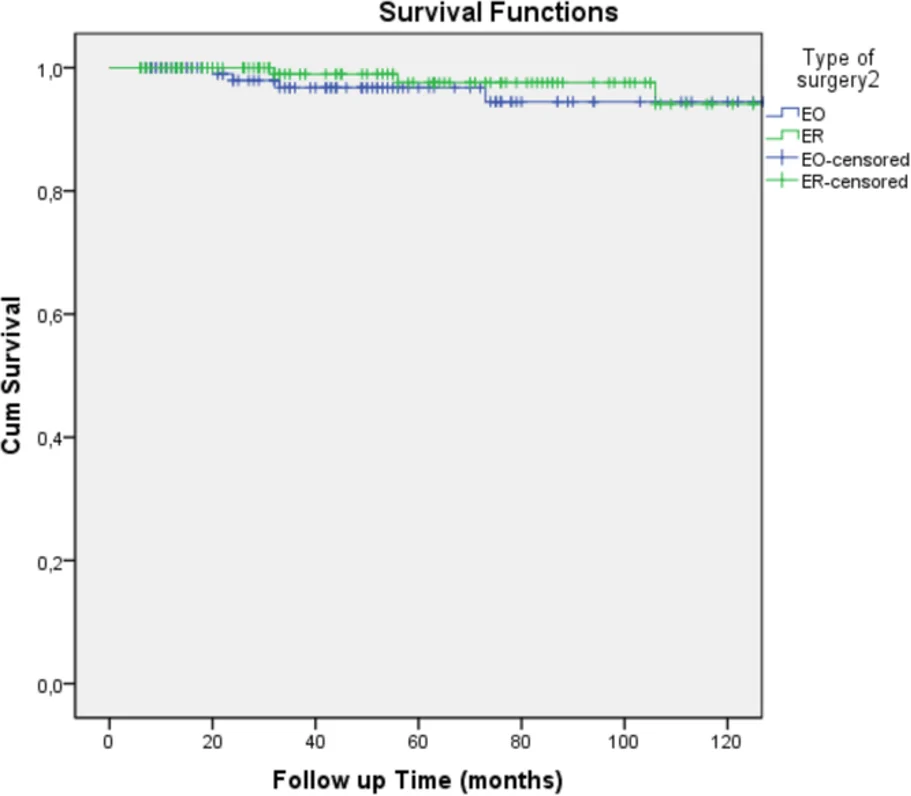
Overall survival curves according to type of surgery. EO, extreme oncoplasty; ER, extreme reconstruction.

**Figure 2:**
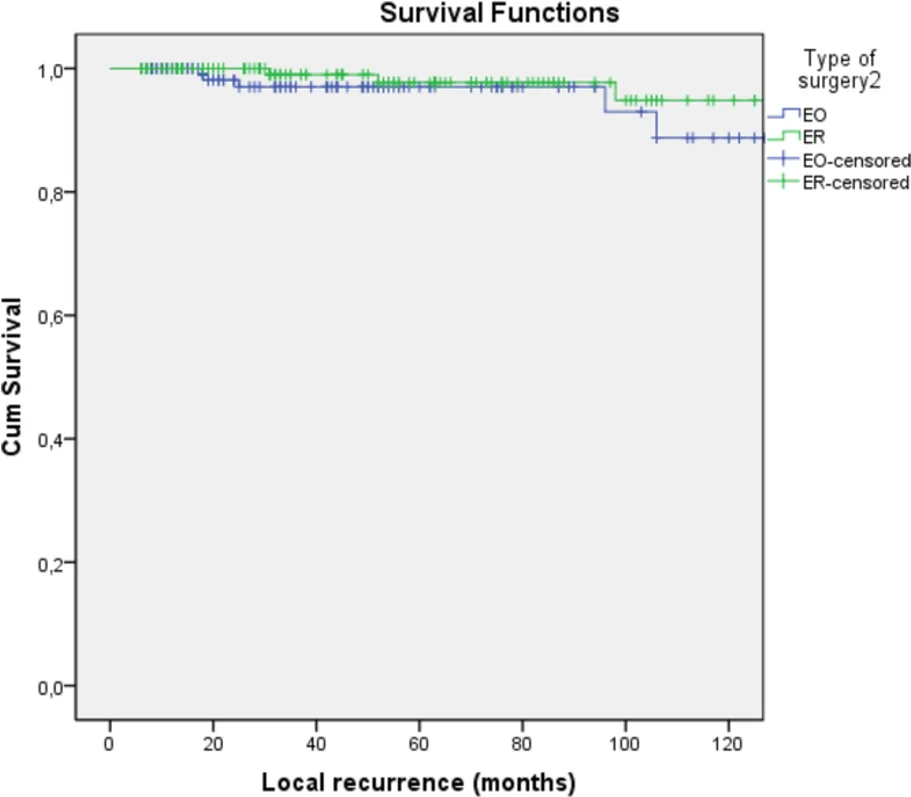
Locoregional recurrence free survival curves according to type of surgery. EO, extreme oncoplasty; ER, extreme reconstruction.

## 4 Discussion

This study represents one of the largest series of EO and utilizes a particularly stringent selection criterion. In Brazil, approximately 40% of breast cancer cases are still diagnosed at stages III and IV, a concerning trend in developing countries, that is particularly prevalent among younger patients [31, 32, 33, 34].

The original definition of EO was a breast conservation operation using oncoplastic techniques, in a patient who, in most physicians' opinions, require a mastectomy. This subjectivity makes comparisons across studies challenging [5, 10, 11, 13, 35, 36]. We preferred to include more objective criteria, including only tumors larger than 5 cm or multicentric tumors [5, 10, 11, 13, 35, 37, 38, 39, 40].

Despite pairing the study groups by age and date of surgery, the EO group exhibited a significantly shorter median follow‐up period compared to the ER group. This disparity may be attributable to systemic barriers, such as difficulties accessing medical assistance within the public health system. Consequently, the limited median follow‐up duration prevents us from drawing accurate, long‐term conclusions regarding outcomes beyond the 5‐year mark.

A recent comparative study by Hossino et al. [13], which examined extreme lesions managed with conservative treatment versus mastectomy, surprisingly reported that none of the patients in their mastectomy group received adjuvant radiotherapy. This contrasts sharply with our study, where 60% of the breast ER group required radiotherapy. Given that most patients with locally advanced breast cancer will necessitate post‐operative radiotherapy, even following a robust response to CT, this factor introduces considerable complexity to breast reconstruction, particularly when utilizing implants and tissue expanders. Consequently, the challenge of post‐mastectomy radiation provides a strong rationale for prioritizing breast conservation in carefully selected cases.

A substantial proportion of patients in the EO group (72.46%) received neoadjuvant CT, which likely contributed to a higher rate of breast conservation. This is noteworthy considering that the majority of patients in the EO group had luminal tumors, which are generally less responsive to neoadjuvant CT. Over the years, there has been an increase in the indication for neoadjuvant CT [41]. In other studies with extreme lesions, the rate of patients undergoing EO who received neoadjuvant CT varied from 6% to 52% [35, 37, 39, 41].

Interestingly, in the EO group, a single surgical procedure was typically sufficient to complete breast reconstruction. In contrast, total breast reconstruction often necessitates multiple surgical stages. Notably, patient and surgeon satisfaction rates remain lower in the total breast reconstruction group despite the increased number of procedures. These findings emphasize the crucial role of comprehensive training and expertise in oncoplastic and reconstructive breast surgery. Selecting the most appropriate surgical approach for each patient requires careful consideration of several factors, including oncological safety (ensuring complete tumor excision with clear margins), patient expectations, and individual aesthetic goals.

Compared with similar series, the outcomes observed in this study were significantly more favorable, the outcomes in this study were significantly favorable. Specifically, our overall re‐excision rate was 6.50%, markedly lower than in the literature average up to 45% [10, 11, 35, 37, 38, 39, 42]. Furthermore, the 2.17% conversion rate to mastectomy due to positive margins fell below the typical reported range of 6% to 13.50% [10, 11, 35, 38, 39, 43]. These favorable outcomes can be attributed to our team's strong belief in the efficacy of BCS and the implementation of a multi‐faceted approach to patient care. This included meticulous preoperative imaging and planning, with pre‐CT tumor localization using skin tattoos, metal clips, or radioactive seeds. Intraoperative frozen section analysis of surgical margins was employed in case of doubt. Furthermore, the surgeons used a diverse repertoire of oncoplastic techniques, including some personal techniques already published, such as classic and disguised geometric compensation mammoplasty and bilobed Lateral Intercostal Artery Perforator (LICAP) flap [20, 44, 45, 46, 47, 48, 49, 50, 51].

Complication rates were significantly higher in the ER group, particularly for major complications requiring re‐intervention. The lower complication rates observed in the EO group may offer several advantages over a total breast reconstruction, such as reduced postoperative morbidity and hospitalization; minimized delays in the initiation of adjuvant therapies, and decreased healthcare costs associated with complications. These factors further support the rationale for pursuing breast conservation whenever feasible.

Satisfaction with breasts and psychosocial well‐being, as measured by the BREAST‐Q, were significantly higher following oncoplastic surgery. A recent meta‐analysis demonstrated that oncoplastic techniques maintain high patient satisfaction rates, even in cases of large or multicentric tumors. This finding underscores the significant role of oncoplastic surgery in preserving patient quality of life while achieving oncologic control [6].

Although the majority of patients in both groups achieved cosmetic outcomes rated as good or excellent according to the Harvard scale and BCCT.core, there were a higher prevalence of poor results in the ER group when assessed using the Harvard scale. A comparative study assessing the reproducibility of different methods for evaluating the cosmetic outcome of breast reconstruction demonstrated that the BCCT.core program exhibited a central bias, tending to classify results as “good” and thus less frequently assigning ratings at the extremes (‘poor’ or ‘excellent’) [29]. In contrast, the Harvard scale, despite its inherent simplicity, showed a superior correlation with patient‐reported opinions, as captured by specialized Quality‐of‐Life scales such as the BREAST‐Q [29]. Given the inherent limitations of current evaluation methods and the absence of a gold‐standard tool for measuring the aesthetic outcome of breast reconstruction, these findings highlight the need for cautious interpretation and suggest the potential benefit of using multiple complementary assessment approaches [29].

In a study by Kilic et al. [52], which included 86 patients with extreme lesions and a median follow‐up of 75 months, the 5‐year overall survival was 90%, while the 5‐year local recurrence‐free survival rate reached 96%. In comparison, our study demonstrated slightly superior outcomes, with a 5‐year overall survival rate of 94.47% and a 5‐year locoregional recurrence‐free survival rate of 97% in the EO group. Previous studies investigating EO have reported local recurrence rates ranging from 2% to 9.10%, 5‐year disease‐free survival rates ranging from 91.50% to 98%, and 10‐year overall survival rates around 88% [5, 10, 35, 37, 39, 52].

This study presents some limitations that should be acknowledged. First, its retrospective nature introduces inherent constraints related to data completeness and control over confounding factors. Selection bias cannot be excluded, as patient inclusion and treatment allocation were not randomized. The follow‐up period was variable among participants, potentially influencing the assessment of long‐term outcomes. Finally, the absence of randomization limits the capacity to draw definitive causal inferences regarding the impact of the reconstructive approach on oncological and aesthetic results.

Patient retention remains a challenge in certain breast units. Specifically, within the public health system, a notable number of patients failed to return for scheduled follow‐up appointments with the reconstructive surgeon within the stipulated timeframe.

## 5 Conclusion

In conclusion, EO is a feasible and safe option for selected patients with large or multicentric breast cancer. Oncologic outcomes are comparable to those of ER. EO provides superior aesthetic results, greater patient satisfaction, and improved quality of life. It is also associated with lower rates of complications. Furthermore, EO requires fewer surgical procedures than mastectomy with reconstruction, underscoring its role as a balanced approach that ensures oncologic safety while enhancing functional and psychosocial outcomes.

These findings support expanding the indications for breast conservation even in large or multicentric tumors. Mastectomy may represent overtreatment for some patients. EO reduces number of procedures, morbidity, and psychosocial impact. Prospective multicenter randomized studies are warranted to validate these results.

## Author Contributions

**Aline R. Nunes‐Reis:** conceptualization, writing – original draft, writing – review and editing, data curation. **Regis Resende Paulinelli:** conceptualization, methodology, formal analysis, writing – review and editing, supervision, data curation. **Rosemar Macedo Sousa Rahal:** writing – review and editing, supervision, data curation. **Luiz Fernando Jubé Ribeiro:** writing – review and editing, supervision, data curation. **Ruffo de Freitas Júnior:** writing – review and editing, supervision, data curation.

## Funding

The authors have nothing to report.

## Conflicts of Interest

The authors declare no conflicts of interest.

## Synopsis

- *Objective*: This study compares extreme oncoplastic surgery with total breast reconstruction in women with large or multicentric breast cancer.
- *Findings*: Oncoplasty achieved equivalent oncologic safety, fewer procedures, lower complication rates, and superior aesthetic outcomes.
- *Conclusion*: Extreme oncoplasty could be a valid alternative to mastectomy with reconstruction, offering better patient satisfaction and quality of life.

## Data Availability

The data that support the findings of this study are available from the corresponding author upon reasonable request.
